# Accuracy of Genomic Predictions Cross Populations with Different Linkage Disequilibrium Patterns

**DOI:** 10.3390/genes15111419

**Published:** 2024-10-31

**Authors:** Lei Jin, Lei Xu, Hai Jin, Shuanping Zhao, Yutang Jia, Junya Li, Jinling Hua

**Affiliations:** 1 College of Animal Science, Anhui Science and Technology University, Chuzhou 233100, China; muy4nj1n@163.com; 2 Anhui Province Key Laboratory of Livestock and Poultry Product Safety Engineering, Institute of Animal Husbandry and Veterinary Medicine, Anhui Academy of Agricultural Sciences, Hefei 230031, China; xuleirock@163.com (L.X.); jinhaizjm@163.com (H.J.); zhaoshuanping@163.com (S.Z.); yutang2018@163.com (Y.J.); 3 Institute of Animal Sciences, Chinese Academy of Agricultural Sciences, Beijing 100193, China

**Keywords:** multipopulation, Chinese indigenous cattle, genome selection, linkage disequilibrium patterns

## Abstract

Background/Objectives: There is a considerable global population of beef cattle, with numerous small-scale groups. Establishing separate reference groups for each breed in breeding practices is challenging, severely limiting the genome selection (GS) application. Combining data from multiple populations becomes particularly attractive and practical for small-scale populations, offering increased reference population size, operational ease, and data sharing. Methods: To evaluate potential for Chinese indigenous cattle, we evaluated the influence of combining multiple populations on genomic prediction reliability for 10 breeds using simulated data. Results: Within-breed evaluations consistently yielded the highest accuracies across various simulated genetic architectures. Genomic selection accuracy was lower in Group B populations referencing a Group A population (*n* = 400), but significantly higher in Group A populations with the addition of a small Group B (*n* = 200). However, accuracy remained low when using the Group A reference group (*n* = 400) to predict Group B. Incorporating a few Group B individuals (*n* = 200) into the reference group resulted in relatively high accuracy (~60% of Group A predictions). Accuracy increased with the growing number of individuals from Group B joining the reference group. Conclusions: Our results suggested that multi-breed genomic selection was feasible for Chinese indigenous cattle populations with genetic relationships. This study’s results also offer valuable insights into genome selection of multipopulations.

## 1. Background

The genome selection (GS) accuracy significantly hinges on the size of the reference population [1]. Notably, GS has seen its most mature application in dairy cows due to their ample reference population [2]. However, challenges arise in the beef cattle industry, distinguished by a large population with numerous small-scale groups. Establishing a distinct reference group for each breed becomes impractical in breeding practice, severely limiting GS application in these groups. To address this, multipopulation/variety GS has emerged, combining reference populations from different breeding organizations, enterprises, or breeds. This approach goes beyond merely adding individuals of the same origin. For a small number of populations, establishing a sufficient reference population size is impossible. Therefore, combining multiple population data for GS increases the reference population size, promotes easy operation, and accomplishes resource sharing, thus making it particularly attractive and practical for small-scale population.

At present, GS has been widely used. Mucha et al. attempted to estimate the genomic estimated breeding value (GEBV) of hybrid progeny using a reference population comprising three dairy goat breeds [3]. GS studies were conducted on single breed and mixed reference populations using Holstein cattle and Jersey cattle. The use of Bayes method resulted in an over 13% increase in the accuracy of GEBV estimation for specific traits in mixed reference populations [4]. Zhou, in comparing GS accuracy using single- and multi-group reference groups from multiple dairy cow groups in Norway, Denmark, Finland, and Sweden, discovered that mixed reference groups enhanced the prediction accuracy of production performance traits for Norwegian Red Bull and Danish dairy cow groups [5]. The Bayes model, coupled with Genome-Wide Association Studies (GWAS) marker screening, proves more suitable for the cost-effective GS of complex traits in multi-variety populations [6]. In the study of multi-breed GS, Van Den Berg et al. suggested that incorporating a marker screening process before the BayesR method could enhance the accuracy of predicting populations not included in the reference population. This marker screening method significantly improved computational efficiency [7]. Brito et al. compared methods of setting verification groups, including generation-based, k-means clustering, genome clustering, and random grouping. Genome clustering demonstrated the highest accuracy, while the k-means clustering method exhibited the lowest accuracy. It is evident that employing a mixed reference population with sufficient numbers yields greater accuracy than that of a single-variety GS [8]. Van den Berg’s study demonstrated the advantages of using sequencing data in conjunction with marker screening methods in multipopulation GS [9].

Simultaneously, studies utilizing SNP chips of varying densities for multipopulation GS research and application have been conducted. In cross-breed GS, incorporating individual data from candidate populations significantly enhances the reference population [10]. The study by Hoze and colleagues additionally demonstrates that utilizing a reference population encompassing multiple breeds leads to a 2.9% improvement in the precision of predictions as opposed to relying on a single-breed reference group [11]. Conversely, Kachman et al. observed that the prediction accuracy, using a mixed reference population from multiple groups, does not surpass that of a single variety in a sufficiently large reference population. Prediction accuracy is higher with close genetic relationships among layers but diminishes when the genetic relationship is distant [12,13]. Currently, GS results in multipopulation scenarios that are unsatisfactory, potentially influenced by genetic relationships, linkage disequilibrium (LD) consistency, and quantitative trait locus (QTL) differences among populations. Thus, conducting multipopulation studies using diverse methods holds significant importance in both GS theory and breeding applications.

Current situationof beef cattle poses a challenge to the efficiency of beef breeding in our country. The genetic resources used in beef cattle production and improvement mainly rely on foreign introductions. Breeding of Chinese beef cattle is in its initial stages, lacking a fully established infrastructure for breeding technology systems, such as performance measurement stations, breeding databases, and genetic evaluation platforms [14]. Since GS technology can make accurate selections without relying on phenotypic and genealogical data, it provides an opportunity for advancing Chinese beef cattle breeding. Chinese indigenous cattle exhibit a diverse pattern of linkage disequilibrium. Consequently, examining these cattle can provide significant insights about the genetic foundations of key characteristics and can be used to evaluate the effectiveness of genomic selection across multiple breeds. The objective of this research is to evaluate the effectiveness of implementing genomic selection across multiple populations in Chinese native cattle and to identify a feasible strategy for genomic selection suitable for Chinese native cattle populations of limited size.

## 2. Methods

The genotype data were retrieved from our previous study [15]. Therefore, no Animal Care Committee approval was necessary for the purposes of this study.

### 2.1. Animals and Methods

All individuals from 10 Chinese cattle breeds (Table 1) were genotyped via the Illumina BovineHD Beadchip (Illumina, Inc., San Diego, CA, USA). The simulation procedure was set up to generate a similar linkage disequilibrium structure of each breed as described by a previous study [15]. We started with 21–26 available samples for each breed comprising 658,234 SNPs. For each breed, we simulated 1500 individuals via resampling, which assumes a block of 500 adjacent markers for each population. Thus, the simulated data can retain similar patterns of LD (broken by strong recombination hotspots) and allele frequencies as observed in the original data.

SNP quality control (QC) involved PLINK v1.9 [16]. Samples with total call rates < 0.90 were excluded, and only autosomal SNPs were considered for subsequent analyses. Samples that had a total call rate of less than 0.90 were filtered out, and for the following analyses, only the autosomal SNPs were taken into account. SNPs with call rates (CRs) < 0.90, minor allele frequencies (MAFs) < 0.01, and significant deviation from Hardy–Weinberg Equilibrium (*p* < 1.0 × 10^−6^) were excluded. After QC, genotype phasing was performed using BEAGLE v5.0 [17]. The Chinese native cattle populations were categorized using K-means clustering, as executed within the R 3.6.1 program [18,19].

### 2.2. Principal Component Analysis and Persistence of Allele Phase

To examine the genetic makeup of both actual and simulated populations, principal components and the genomic relationship matrix (GRM) [20] were computed utilizing high-quality SNPs. Principal components were estimated using the prcomp function implemented in R package “stats”.

The continuity of alleles between the real and simulated genotypes was evaluated, with phase consistency being quantified by determining the Pearson correlation coefficient of the average linkage phase across various distances. The correlation coefficients (r) were calculated for marker pairs among populations, categorizing the marker distances into specific intervals: 2.5 kilobases (kb) for the short-range category (0–10 kb), 10 kb for the medium-range (10–100 kb), and 100 kb for the long-range (100–1000 kb).

### 2.3. Simulation

Phenotypes were simulated corresponding to the simulated genotype. A variety of scenarios were emulated as outlined in Table 2, encompassing different levels of heritability, quantities of QTLs, and distributions of QTL effects. A selection of SNP markers were randomly designated as QTLs, with their additive impacts being drawn from three distinct normal distribution: N (0, 0.0001 $\sigma_{g}^{2}$), N (0, 0.001 $\sigma_{g}^{2}$), and N (0, 0.01 $\sigma_{g}^{2}$), which present large-, medium-, and small-effect QTLs, respectively, and $\sigma_{g}^{2}$ is the additive genetic variance.

The true breeding values are ascertained by calculating the sum of the effects of their genotypes on the QTL. Environmental impacts were assigned randomly from a normal distribution characterized by a mean of 0 and a variance given by the formula $\frac{V_{g}\left(1-h^{2}\right)}{h^{2}}$, with $V_{g}$ representing the genetic value variance and $h^{2}$ representing the trait heritability. The individual phenotypes were derived from the combined effects of genetics and environment.

For each scenario, phenotypes were simulated, where residuals were extracted from a suitable Gaussian distribution to generate three traits, each possessing a heritability of 0.1, 0.3, and 0.6, respectively. Each of these scenarios were replicated 10 times.

True breeding values (TBVs) were estimated by aggregating the impacts of the genotypes at the QTLs, as dictated by the following formula:

(1) $$TBV=\sum_{j=1}^{n}x_{ij}a_{j}$$

where $x_{ij}$ is the genotype of individual j coded as 0, 1, and 2 for QTL i; $a_{j}$ is the additive effect of QTL *i*; and n is the number of QTLs.

### 2.4. Genomic Evaluation

Genomic breeding values were calculated across all scenarios employing the method of genomic best linear unbiased prediction (GBLUP). The GBLUP model was applied using the following formula:

(2) $$y=X_{b}+Z_{a}+G_{g}+e$$

where y represents a vector of observed phenotypes. The matrices X, Z, and G are used to allocate the phenotypes to the vectors b, a, and g, respectively, which correspond to fixed effects (such as the overall mean and breed-specific effects) and polygenic breeding values derived from genomic information. The vector e is a vector of residual errors distributed as N (0, I $\sigma_{e}^{2}$), following the identity matrix I and error variance $\sigma_{e}^{2}$. Polygenic and genomic breeding values were distributed according to normal distributions, denoted as N (0, A $\sigma_{a}^{2}$) and N (0, GRM $\sigma_{g}^{2}$), respectively, where A represents the numerator relationship matrix, $\sigma_{a}^{2}$ corresponds to the additive genetic variance, the GRM stands for the genomic relationship matrix, and $\sigma_{g}^{2}$ refers to the genetic variance attributed to genomic variants. The GRM was constructed as described previously [21].

### 2.5. Reference and Validation Populations

Three scenarios of references were examined, which varied based on the size and composition of the reference population.

Scenario I: in this case, the reference population was constituted by 1200 individuals drawn from a single simulated breed, with each breed representing a unique reference population.

Scenario II: the reference population, consisting of 1200 individuals, was selected at random from three distinct simulated breeds, ensuring an equal representation from each breed’s population. K-means clustering and Principal Component Analysis (PCA) were applied to categorize the ten populations into three groups. These groups were then merged to create three separate reference populations. Additionally, for comparative analysis, a fourth reference population was established, which included three breeds (XZC, LSC, and HNC) from the different groups.

Scenario III: a reference population was established by pooling individuals from 10 different breeds, achieved by selecting 120 individuals at random from each of the ten individual populations.

The accuracy and bias of genomic prediction (GP) were assessed using a 5-fold cross-validation (CV) procedure. Within this methodology, the entire population was randomly partitioned into five distinct groups at random. During each round of the process, one group was designated as the validation set, and the other four groups functioned as the reference set. This random division was conducted five separate times for both the GBLUP and Bayesian models. The accuracy of the predictions was assessed by computing the average Pearson correlation coefficient between the adjusted phenotypic values and the GEBVs (genomic estimated breeding values) for the validation groups [22]. The formula is as follows:

(3) Prediction accuracy=cor(y, gebv)

where y denotes the vector of adjusted phenotypes, and gebv represents the vector of GEBVs.

To quantify the extent of prediction inflation or deflation, we calculated the correlation coefficient between the adjusted phenotype and GEBVs for individuals within the validation group. This metric was derived as follows:

(4) $$b=\frac{cor(y,\;gebv)}{var(gebv)}$$

where y and gebv are the same as that in Equation (3), with the regression coefficient for unbiased models anticipated to be approximately 1, whereas values > 1 indicate a biased deflation prediction of GEBVs, and values < 1 indicate a biased inflation prediction of GEBVs [23].

To ascertain if haplotype-based approaches could markedly enhance the prediction accuracy compared to SNP-based methods, a one-sided paired *t*-test was employed to assess the statistical significance of the observed differences. The threshold for statistical significance was established at a *p* < 0.05.

### 2.6. Comparison of Genome Prediction Accuracy of Different Genetic Relationships Across Populations

Table 2 outlines the 11 selected combinations, employed to assess genome prediction accuracy. Using a reference population of 400 individuals from population 1 genome breeding values were predicted for 200 individuals each from population 1 and population 2. The accuracy was evaluated using the following formula:

$$r_{GEBV}=Cor(GEBV,EBV)$$

The precision of genomic prediction was gauged by the correlation between the projected genetic values and the true breeding values of the synthesized phenotypes. Each simulation scenario was conducted in quintuplicate, and the average accuracy was determined from these replicates.

### 2.7. Assess Genetic Relationships Between Populations

To fulfill genetic assessment requirements, 70 individuals were selected to maximize genetic relationships. Chip genotype data typing were utilized for this purpose, ensuring accuracy in assessing LD levels, as >70 samples are needed for a reliable evaluation of LD structure consistency between the two populations.

## 3. Results

### 3.1. Genetic Relationships Between Populations

The genetic relationship among 10 simulated populations was evaluated. For ease of quantification, we assessed LD structure consistency between the populations, ensuring marker spacing within the 0–100 mb range. A representative sample of 11 combinations (Table 3) was selected to evaluate the accuracy of cross-species genome selection.

Results indicated LD structure consistency ranging from 0.107 to 0.516. The lowest LD structure consistency (0.107277) was observed between simnd and simyh, while the highest (0.516203) was between simls and simzt. We attribute this variation to the correlation between LD structure consistency and individuals from the 10 Chinese cattle breeds. As the distance increases among individuals from these breeds, LD structure consistency between populations also increases.

### 3.2. Multipopulation Genomic Genetic Assessment of Variety Combinations

**Figure 1:**
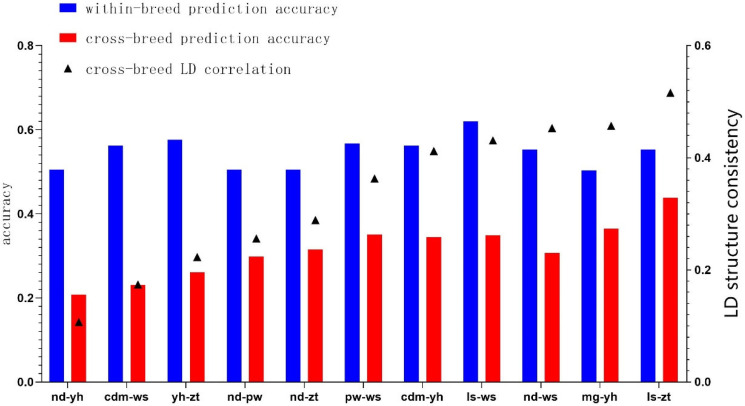
Relationship between cross-breed genome prediction accuracy and LD consistency between populations.

Genetic relationships guided the selection of populations for cross-breed and joint genetic assessment. Populations with LD structure consistency > 0.45 (marker spacing within 0–100 mb) were suitable for combined genomic genetic assessment. Figure 1 illustrates that, with a reference population comprising 400 and 200 individuals from populations A and B, respectively, the prediction accuracy of the genomes increased as LD consistency improved between the populations.

After comparison, the average prediction accuracy of four different simulated phenotypes was shown in Table 4 and Figure 1. Overall, cross-breed genome prediction accuracy rises with increased relationship values. When LD structure consistency between populations exceeds 0.45, cross-breed genome prediction accuracy for low, medium, and high genetic traits can reach 0.30, 0.35, and 0.37, respectively, equivalent to 60–69% of single-breed genome prediction accuracy.

### 3.3. Multipopulation Genomic Genetic Assessment Methods

**Figure 2:**
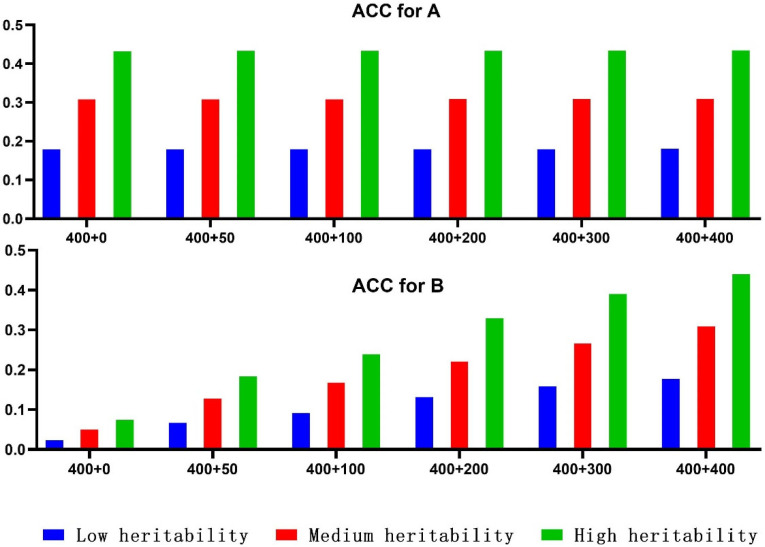
Influence of different reference population settings on cross-breed prediction accuracy (LD correlation between AB is 0.5).

Figure 2 illustrates the average accuracy of predictions for group A, indicated as 0.18, 0.31, and 0.43 for heritability (h^2^) of 0.1, 0.3, 0.6. Regardless of heritability, the prediction accuracy remains relatively stable as the number of individuals in group B increases. However, when the LD correlation between group A and B is high (0.5), utilizing the reference group of group A (*n* = 400) to predict group B results in low accuracy. When a few individuals of Group B (*n* = 200) are added to the reference group, the accuracy is relatively high (~60% of the prediction of group A). Furthermore, the accuracy increased as the number of individuals joining Group B increased.

When the same number of individuals joined Group B, the accuracy improvement varied with increasing heritability. Even in direct cross-breed genome assessments between two closely related breeds, the accuracy remains low. In practical applications, when using variety A to predict the genome of variety B, adding an appropriate number of variety B individuals to the reference population is essential for obtaining reliable accuracy.

## 4. Discussion

### 4.1. Simulation of Genotype and Phenotype

The assessment of multipopulation genomic prediction is contingent upon the LD patterns present within these populations.

Consequently, comprehending the LD patterns across various populations can provide valuable insights for exploring the genomic prediction accuracy in a multipopulation context. In the current investigation, simulations were conducted using a resampling method to accurately capture and reflect the allele frequencies and population-specific LD patterns observed in actual populations [24,25]; the genotype for each simulated individual was generated by extracting and repurposing genotype segments from the authentic genotypes of the animals under study. Consequently, the simulated population maintains the fundamental LD patterns structures and allele frequencies that are characteristic of the genuine data from Chinese indigenous cattle. The findings from our study serve as significant validation for the theoretical assessment of genomic prediction in Chinese indigenous cattle breeds.

Additionally, we assessed the characteristics of genomic variation across the entire genome and compared the efficacy of various strategies [26]. Based on the outcomes of PCA and the continuity of phase persistence assessments, it was determined that the simulated genotypes are capable of mirroring the realistic LD patterns, thereby making them suitable for exploring genomic prediction across multiple breeds.

In the simulation process, QTLs were chosen at random from the SNP loci present in the genuine genotype dataset. The allele frequencies of these QTLs vary across different breeds, suggesting that the majority of QTLs are segregating within these breeds. The phenotypes simulated for individuals within each population were distinct due to variations in MAF, which aligns with the genetic diversity observed in real datasets across different populations. This approach is instrumental in assessing the efficacy of genomic prediction for multiple populations.

### 4.2. LD Level and LD Structure Consistency

To assess LD levels in populations, this study utilized r^2^ instead of *D*′ due to its susceptibility to sample size [27]. Khatkar’s study indicated that an accuracy of 0.85 is achievable with a sample size > 55, which the groups/populations in this study met, ensuring sufficient LD assessment accuracy. Furthermore, LD levels, as observed in other breeds like Angus and Holstein cattle, decrease with increased marker distance [27,28,29].

The GP accuracy is influenced by the heritability and genetic structure of traits, impacting genetic progress in breeding [5,30]. GEBV accuracy is affected by trait heritability, with lower heritability leading to lower prediction accuracy [31,32,33]. In the simulation study of Spanish regional cattle, Mouresan et al. determined that gene prediction accuracy for a trait with 0.4 heritability ranged between 0.363 and 0.330 in the mixed seven breeds. Additionally, prediction accuracy decreased with decreasing heritability [34]. Esfandyari et al. [35] explored the advantages of applying GS to purebreds for enhancing the performance of crossbred performance (CP), using purebred data under two scenarios: low or high linkage disequilibrium (LD) phase correlation between the two pure lines. Their findings suggest that when there is a high correlation of LD phase between both pure lines, combining them into a single reference population enhances the selection accuracy of selection for predicting marker effects. Morgante F et al. [36] showed that within a population of individuals without genetic relationships and with low levels of LD, the additive GRM, which was built from all common variants (~1,800,000), failed to provide adequate predictive accuracy. This was true irrespective of the trait’s genetic architecture, including instances where the trait exhibited a purely additive architecture. Simultaneously, Cañas-Álvarez et al. [37] emphasized that all average accuracy estimates were positive, aligning with the persistent LD found between these populations and their genetic closeness.

### 4.3. Predictive Accuracies from Admixed Population

In this study, we noted that predictive accuracies were comparatively modest when assessing the reference and validation populations derived from distinct breeds. This discrepancy may arise due to high LD within the studied breeds, contributing to correlation between SNPs and causal polymorphisms, unlike in other breeds [38]. These findings align with prior empirical studies on traits of comparable heritability [31,32,39]. Meuwissen et al., through a simulation analysis, determined that a predictive reliability of 0.62 could be achieved for a training set comprising 1000 phenotypes with a heritability estimate of 0.5 [31]. The composition of the reference population significantly influenced the precision of the predictions, especially concerning the level of genetic association between the reference and the validation populations [40]. Our study indicates that incorporating these breeds into the reference population enhances the accuracy of predictions. Notably, genomic prediction accuracy, reported in a previous study for Holstein–Friesian and Jersey cows, ranged from 0.01 to 0.19.The addition of individuals from other breeds did not significantly enhance accuracy in the reference population [41]. In application, merging data from different breeds can augment genetic progress when the components of the mix share genetic ties. The precision of predictions was enhanced through the integration of multiple breeds grouped via the K-means approach [15]. Our results provided valuable insights into applying a pooled data approach for multiple population selection. However, certain investigations have indicated that the strategy of pooling data could potentially reduce the predictive precision for mixed populations [42,43].

Our results corroborated earlier research, demonstrating that when employing a composite reference of seven breeds, the predictive accuracies for Spanish native cattle with a heritability of 0.4 varied from 0.363 to 0.330. It is important to note that these accuracies tend to diminish as heritability decreases [34]. Utilizing a pooled data strategy could result in reduced accuracy, especially for elements of admixed populations with limited population sizes [44]. The primary concern revolves around optimizing benefits while considering genotyping costs. To improve the precision of genomic breeding value predictions, it is essential to include a significant number of animals with known genotypes and phenotypes in the training dataset [31,45]. Incorporating individuals from populations with genetic affinities is advantageous for the genotyping of smaller populations. This methodology is practical for the implementation of genomic prediction across a variety of small breeds, including local cattle breeds in different nations. This study revealed lower accuracy in cross-breed predictions, where the reference and validation populations originated from different breeds. This reduced accuracy may stem from the high LD [between QTL and single-nucleotide polymorphism (SNP)] present in the reference population but not necessarily mirrored in the validation population. Similar trends have been observed in other populations [32,38,39]. The composition of the reference population significantly impacts the precision of estimating genome breeding values, particularly when there is a considerable genetic distance between the reference and validation populations [40]. In breeding scenarios, the primary concern revolves around achieving accurate selection results while managing breeding costs. To enhance the accuracy of genomic breeding value estimation, it becomes imperative to establish a larger reference population incorporating both genotype and phenotypic data [45].

### 4.4. Effect of Heritability and Genetic Architecture

The trait’s heritability and genetic framework can affect the genetic improvement achieved through genomic prediction within breeding initiatives [5,30]. In our investigation, GBLUP was employed to forecast genetic merit, operating under the premise that each marker exerts an equal influence [46]. The heritability of a phenotype is a determinant of the dependability of GEBVs [31,33,47], with traits exhibiting low heritability generally yielding lower predictive accuracies. In our simulation, reduced heritability predominantly led to diminished predictive precision for the majority of traits and situations examined. The additive model, which includes all common variants, was only able to explain a fraction of the total heritability forcomplex trait [36], the observed decline in predictive accuracy in actual data can be ascribed to the “missing heritability” issue, stemming from the impact of non-additive genetic factors.

Previous research has indicated that genomic predictions derived from real data were not aligning consistently with the outcomes of simulation analyses [48,49]. A potential explanation is that the simulated data, which encompass a diverse genetic structure, substantially deviate from the characteristics of actual populations. Numerous investigations have contrasted methodologies using simulated genetic frameworks that include 50 or fewer QTLs, and their conclusions have demonstrated that the genetic architecture can influence the precision of predictions. This includes the number of QTLs and the variance they contribute [48,50]. In this study, individuals from 10 Chinese cattle breeds are employed, and three different strategies simulate and evaluate genetic relationships among the 10 populations. Thus, results indicate the feasibility of a multipopulation GS strategy for Chinese native cattle, even under small population sizes.

## 5. Conclusions

In conclusion, this study emphasizes that the accuracy of cross-breed genome assessments is notably low when conducted between closely related breeds. However, a practical and effective measure to improve accuracy in genome selection for breeds with small populations involves integrating individuals from other breeds with a close genetic correlation to the reference population. Upon establishing the combination method for genetic evaluation across multipopulations, we utilized common LD information to filter markers. The procedure involved the following steps: (1) calculating LD levels individually for markers in the genotype data of each variety; (2) within each variety, sorting the *r_ij_* values among markers and selecting markers based on the criteria of 0.80 < $r_{ij}$ < 0.999 or 0.90 < $r_{ij}$ < 0.999; (3) by retaining a pair of markers in all populations, the two SNPs were designated as SNP sets, forming the basis for interpopulation LD selection for multipopulation genome genetic assessment. This finding holds crucial practical significance for implementing GS in local beef cattle breeds across diverse countries. This study’s results also furnish valuable references for optimizing genome selection strategies in multipopulation contexts.

## Figures and Tables

**Table 1:** Phenotypic simulation of the information on breeds.

Cattle Breeds	Abbreviation	Number
Inner Mongolia cattle	MGC	21
Yanhuang cattle	YHC	24
Caidamu cattle	CDM	25
Xizang cattle	XZC	26
Pingwu cattle	PWC	24
Liangshan cattle	LSC	22
Zhaotong cattle	ZTC	23
Wenshan cattle	WSC	24
Hannan cattle	HNC	26
Nandan cattle	NDC	25

**Table 2:** Phenotypic simulation strategy.

Phenotypic Simulation Strategy	nQTL ^1^	nS ^2^	nM ^3^	nL ^4^	Heritability (h^2^)
Strategy I	100	0	0	100	0.1/0.3/0.6
Strategy II	2000	1361	614	25	0.1/0.3/0.6
Strategy III	5000	4595	390	15	0.1/0.3/0.6
Strategy IV	10,000	10,000	0	0	0.1/0.3/0.6

^1^ Total number of QTLs. ^2^ Number of QTLs with small effect (nS). ^3^ Number of QTLs with medium effect (nM). ^4^ Number of QTLs with large effect (nL).

**Table 3:** LD structure consistencies were selected between cultivar combinations for comparison of cross-breed genome predictions.

Population 1	Population 2	LD Structure Consistencies
simnd	simyh	0.107277
simcdm	simws	0.174347
simyh	simzt	0.2228
simnd	simpw	0.255829
simnd	simzt	0.28869
simpw	simws	0.363149
simcdm	simyh	0.411982
simmg	simyh	0.457195
simls	simws	0.431238
simnd	simws	0.453237
simls	simzt	0.516203

Simmg (Simulated Inner Mongolia cattle), Simyh (Simulated Yanhuang cattle), Simcdm (Simulated Caidamu cattle), Simpw (Simulated Pingwu cattle), Simls (Simulated Liangshan cattle), Simzt (Simulated Zhaotong cattle), Simws (Simulated Wenshan cattle), and Simnd (Simulated Nandan cattle).

**Table 4:** Comparison of genome prediction accuracy of different genetic relationships across populations.

Pop	cor100	Low Heritability		Medium Heritability		High Heritability	
		Population 1	Population 2	Population 1	Population 2	Population 1	Population 2
nd-yh	0.107	0.478	0.167	0.493	0.191	0.505	0.208
cdm-ws	0.174	0.582	0.184	0.565	0.212	0.562	0.231
yh-zt	0.223	0.556	0.207	0.588	0.238	0.576	0.261
nd-pw	0.256	0.479	0.234	0.494	0.270	0.505	0.298
nd-zt	0.289	0.478	0.248	0.494	0.287	0.505	0.315
pw-ws	0.363	0.558	0.274	0.581	0.317	0.567	0.351
cdm-yh	0.412	0.582	0.274	0.564	0.318	0.562	0.345
ls-ws	0.431	0.705	0.281	0.647	0.326	0.620	0.349
nd-ws	0.453	0.516	0.259	0.553	0.301	0.553	0.307
mg-yh	0.457	0.479	0.298	0.491	0.347	0.503	0.365
ls-zt	0.516	0.519	0.347	0.550	0.404	0.553	0.438

cor100 refers to the inter-marker LD structure consistency between two populations in the range of 0–100 mb; low, medium, and high forces represent heritability of 0.1, 0.3, and 0.6, respectively.

## Data Availability

The raw data cannot be made available, as they are the property of the cattle producers in China, and this information is commercially sensitive.

## Abbreviations

The following abbreviations are used in this manuscript:

GS: genome selection; GEBV: genomic estimated breeding value; GWAS: genome-wide association studies; LD: linkage disequilibrium; QTL: quantitative trait locus; h2: heritability; CP: crossbred performance; SNP: single-nucleotide polymorphism; Simmg: Simulated Inner Mongolia cattle; Simyh: Simulated Yanhuang cattle; Simcdm: Simulated Caidamu cattle; Simpw: Simulated Pingwu cattle; Simls: Simulated Liangshan cattle; Simzt: Simulated Zhaotong cattle; Simws: Simulated Wenshan cattle; Simnd: Simulated Nandan cattle.
